# Invertebrate Iridoviruses: A Glance over the Last Decade

**DOI:** 10.3390/v10040161

**Published:** 2018-03-30

**Authors:** İkbal Agah İnce, Orhan Özcan, Ayca Zeynep Ilter-Akulke, Erin D. Scully, Arzu Özgen

**Affiliations:** 1 Department of Medical Microbiology, School of Medicine, Acıbadem Mehmet Ali Aydınlar University, Atasehir, Istanbul 34752, Turkey; orhn.ozcn@hotmail.com (O.Ö.); aycazeynep@gmail.com (A.Z.I.-A.); aozgen@gelisim.edu.tr (A.Ö.); 2 Department of Biostatistics and Medical Informatics, Acıbadem Mehmet Ali Aydınlar University, Atasehir, Istanbul 34752, Turkey; 3 Evolutionary Genetics, Groningen Institute for Evolutionary Life Sciences, University of Groningen, P.O. Box 11103, 9700 CC Groningen, The Netherlands; 4 Stored Product Insect and Engineering Research Unit, USDA-Agricultural Research Service, Center for Grain and Animal Health Research, Manhattan, KS 66502, USA; erin.scully@ARS.USDA.GOV; 5 Vocational School of Health, Istanbul Gelisim University, Avcılar, Istanbul 34310, Turkey

**Keywords:** invertebrate iridoviruses, genomics, proteomics, classification

## Abstract

Members of the family Iridoviridae (iridovirids) are large dsDNA viruses that infect both invertebrate and vertebrate ectotherms and whose symptoms range in severity from minor reductions in host fitness to systemic disease and large-scale mortality. Several characteristics have been useful for classifying iridoviruses; however, novel strains are continuously being discovered and, in many cases, reliable classification has been challenging. Further impeding classification, invertebrate iridoviruses (IIVs) can occasionally infect vertebrates; thus, host range is often not a useful criterion for classification. In this review, we discuss the current classification of iridovirids, focusing on genomic and structural features that distinguish vertebrate and invertebrate iridovirids and viral factors linked to host interactions in IIV6 (Invertebrate iridescent virus 6). In addition, we show for the first time how complete genome sequences of viral isolates can be leveraged to improve classification of new iridovirid isolates and resolve ambiguous relations. Improved classification of the iridoviruses may facilitate the identification of genus-specific virulence factors linked with diverse host phenotypes and host interactions.

## 1. Introduction

**Figure 1:**
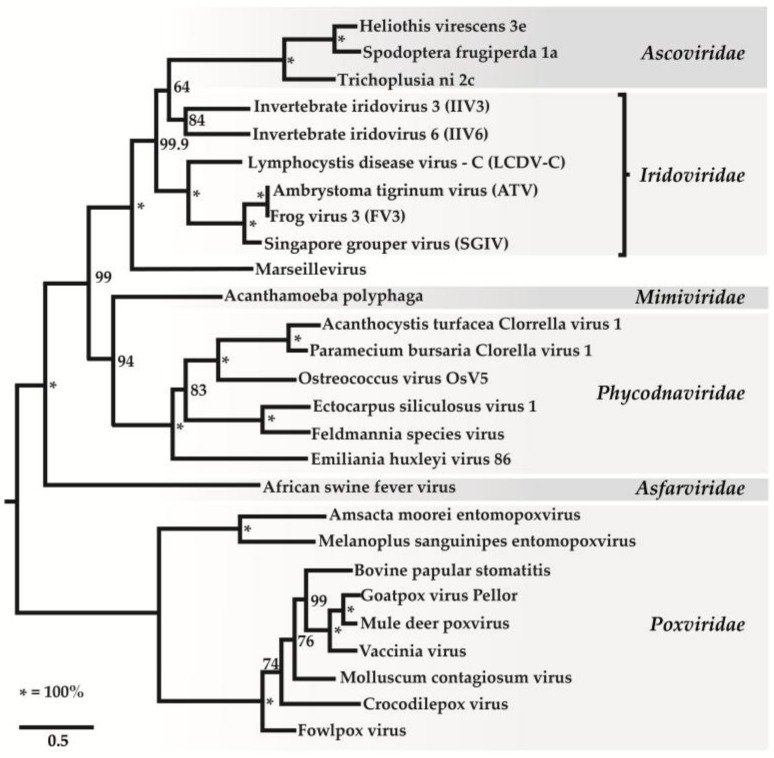
Phylogenetic relationships of iridovirids with other nucleocytoplasmic large DNA viruses A maximum-likelihood tree constructed based on concatenated alignments of 1849 positions of five NCLDV core proteins: A1L/VLTF2 transcription factor, A32 ATPase, D5 type ATPase, DNA polymerase B, and major capsid protein using the TreeFinder program with the estimated site rates heterogeneity and with the WAG (Whelan and Goldman) substitution model. The expected-likelihood weights of 1000 local rearrangements were used as confidence values of TreeFinder tree branches. Scale bar represents amino acid substitutions per site (This figure was used with permission from PNAS [8] and ICTV [4] where it was presented earlier and slightly modified its presentation).

**Figure 2:**
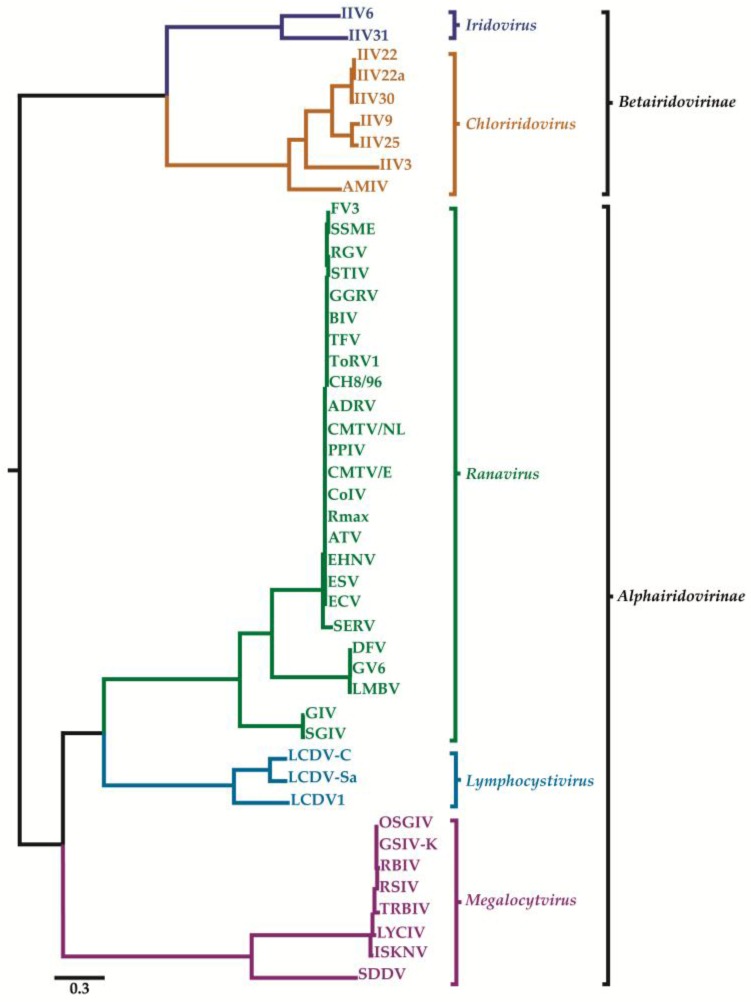
ML analysis based on 26 core genes conserved among all members of the family *Iridoviridae* (iridovirids). The phylogram shows a clear division of genera within the *Alphairidovirinae* (*Ranavirus*, green; *Lymphocystivirus*, cyan; *Megalocytivirus*, purple) and *Betairidovirinae* (*Iridovirus*, blue; *Chloriridovirus*, brown) subfamilies. Virus species and isolates are indicated by abbreviations, listed in Table 2. Members of accepted species are shown in bold typeface. The tree was constructed using maximum likelihood analysis using IQ-TREE open source software [9] under optimum substitution model LG+I+G4 according to Bayesian Information Criterion (BIC) [10].and the concatenated amino acid (aa) sequences of 26 core genes (19,773 aa characters including gaps) from 45 completely sequenced iridovirus genomes. The tree was midpoint rooted and branch lengths are based on the number of inferred substitutions, as indicated by the scale bar. The branch points that separate the iridovirid genera are supported by bootstrap values greater than 99%. For other branch points all bootstrap values are >70% except for those displaying high levels of amino acid similarity, e.g., TFV vs. BIV/GGRV, 66%; PPIV vs. CMTV/2013/NL et al., 49%; TRBIV vs. RSIV et al., 57%; and LYCIV vs. TRBIV et al., 56%. Scale bar represents amino acid substitutions per site. This figure was published as an ICTV report [1,2].

Iridovirids are nucleocytoplasmic large dsDNA viruses (NCLDVs) that can be divided into five genera: *Ranavirus, Lymphocystivirus, Megalocytivirus*, *Iridovirus*, and *Chloriridovirus* (Figure 1 and Figure 2; Table 1 and Table 2) under two sub-families defined recently as *Alpha*- and *Betairidovirinae* by ICTV (International Committee on Taxonomy of Viruses) [1,2]. Recently, the term “iridovirids” was implemented rather than using general term iridoviruses to family members to avoid confusion between members of the genus *Iridovirus* and members of the family with the same name. In this review, all members of the family will be referred to as “iridovirids”, members of the genus will be referred to as “iridoviruses”, and the abbreviation IIV will refer to both genera of invertebrate iridoviruses (*Iridovirus* and *Chloriridovirus*). The iridovirids were initially distinguished from one another according to their virion particle size, host preference, presence of a DNA methyltransferase gene, GC content and phylogeny based on the amino acid sequence of the major capsid protein [3,4]; however, phylogenetic analysis based on complete genome sequences now provides a superior method of differentiation and classification (Figures S1 and S2) and possibly using pan-genomic data will advance the analysis of viral phylogenies, which may ultimately lead to the development of new classification criteria on virus classification in general.

New iridovirids are continuously being discovered; however, limited number of IIVs have been sequenced, and thus, it is hard to precisely quantify or estimate the genetic heterogeneity and variability within each genus. In addition, it is still not clear what type of information/criteria would be sufficient to determine whether newly discovered iridovirids represent new species or whether these new isolates are just variants or strains derived from an existing species. A comprehensive list of identified iridovirids with completed genome sequences is shown in Table 2. Within the genus *Iridovirus*, two species have been identified: Invertebrate iridescent virus 1 (IIV1), which was the first IIV species isolated and infects the soil-dwelling European crane fly *Tipula paludosa* (Diptera) [5] and Invertebrate iridescent virus 6 (IIV-6), also referred to as Chilo iridescent virus (CIV), which was isolated from diseased larvae of the rice stem borer, *Chilo suppressalis* (Lepidoptera; Pyralidae) in Japan [6]. At least two strains of IIV6 have been identified and are routinely used in laboratories around the world and an IIV-6 isolate from Germany was completely sequenced [7]. However, the genomic differences between this isolate and the isolates commonly used in New Zealand, Australia, and the USA laboratories have not yet been investigated. The objective of the review is to discuss the status of current research on this interesting but poorly understood family of isometric DNA viruses and will focus primarily on describing molecular studies on genome organization, gene expression strategies, virion proteins, and relatedness of the IIVs using the best-studied virus, IIV-6, as the primary model.

## 2. Classification of Iridovirids

Using a maximum likelihood-based phylogenetic analysis on a concatenated sequence of 26 core genes present in all 45 iridovirid genome sequences, four major clades were apparent, with members of the genera *Ranavirus, Lymphocystivirus* and *Megalocytivirus* (vertebrate iridoviruses) forming three strongly supported monophyletic clades. The genera *Iridovirus* and *Chloriridovirus* (IIVs) formed a monophyletic clade, but the two genera diverged significantly from one another, as indicated by the relatively long branch lengths on this node (Figure 2). Due to the independent monophyletic origins of vertebrate (*Ranavirus*, *Lymphocystivirus*, and *Megalocytivirus*) and invertebrate iridoviruses (*Iridovirus* and *Chloriridovirus*), two new subfamilies have been proposed. The first subfamily (*Alphairidovirinae*) encompasses all iridoviruses whose hosts are ectothermic vertebrates (fish, reptiles, and amphibians) and the second family (*Betairidovirinae*) contains iridoviruses that infect invertebrates (e.g., insects, crustaceans, etc.). This latter subfamily contains the *Iridovirus* and *Chloriridovirus* genera. These new classifications were discussed in the recent ICTV study group on family *Iridoviridae* (Figure 2) [1,2].

Previously, three major clades were defined within the genus *Iridovirus* using a molecular analysis of restriction endonuclease (REN) profiles of 16 iridovirid genomes as well as *mcp* amplicons combined with DNA hybridization and dot blots by Williams and Cory [56]. Later Webby and Kalmakoff performed molecular analysis of *mcp* genes (formerly gene named as *msp*) [57], terminal redundancies and DNA-DNA homologies [58]. As a result of both studies, three major groups in genus *Iridovirus* were identified: custaceoiridovirus (group 1), oligoiridovirus (group 2), and polyiridovirus (group 3). Group 1 comprised IIV-31 and Popillia japonica IV [56] whereas group 2 included IIV-6 and IIV-21. Group 3 was the largest group, which contained nine different species; IIV-1, IIV-21, IIV-22, IIV-23, IIV-24, IIV-25, IIV-29, IIV-30 and Anticarsia gemmatalis IV.

However, a more recent study performed by Wong et al. [13] using 26 iridovirid core genes indicated that IIV-3 was more closely related to the IIVs than previously estimated. IIV-3 showed a closer relationship to the group 3 polyiridovirus complex when compared to IIV-6, which belongs to the oligoiridovirus complex, group 2 [13]. In addition, recent MCP protein sequence-based phylogenetic information from mosquito iridescent viruses from *Culex pipiens* [59] and *Anopheles minimus* [51] also supported the phylogenetic proximity of the group 3 polyiridovirus viruses to IIV-3.

The iridovirid proteins D5 helicase primase, DNA packaging ATPase (A32), viral late transcription factor 3 (A2L/VLTF3), major capsid protein (MCP) and subfamily B viral DNA polymerase show high amino acid similarities when compared to other nucleocytoplasmic large DNA viruses (NCLDV), especially in the families *Ascoviridae*, *Phycodnaviridae*, *Marseilleviridae*, *Mimiviridae*, *Poxviridae*, Iridoviridae and Asfarviridae [4,8]. Based on a phylogenetic analysis using genes conserved among NCLDVs, the proposals of Colson et al. [60] were supported: (1) all iridovirids share a common ancestor and (2) NCLDVs should be assigned to a new viral order, the Megavirales [60,61].

This analysis also supported the close relationship between ascoviruses and IIVs, suggesting that ascoviruses emerged recently and share a common ancestor with IIV-6 and IIV-3 [62]. In addition, the study indicated that IIV-6 and IIV-31 and IIV-3 and IIV-9 each formed monophyletic groupings, suggesting that IIV-6 and IIV-31 and IIV-3 and IIV-9 are more closely related than previous studies indicated [62]. In light of these recent findings, the current IIV classification needs to be revised.

Phylogenetic analysis based on MCP sequences [63] is outdated and the more current and accepted approach is to use the conserved iridovirid genes (26 genes) in phylogenetic reconstructions [1,2,13,64]. Whole genome sequences of iridovirids are becoming more common and phylogenetic reconstructions based on whole genome sequences may provide additional resolution in comparison to approaches that use only a subset of the genes. For example, phylogenetic analysis based on complete genome sequences of 50 members of the family *Iridoviridae* (for virus abbreviation see Table 2) was performed using a pipeline created in Geneious R11. Two marseillevirus (Cannes 8 virus; KF261120 and Lausannevirus; NC015326) and three ascovirus (DpAV4a; NC011335, SfAV1a; NC004361, TnAV6a; NC008518) genomes were included as outgroups. First, the sequence directions were rearranged so that all genes were oriented in the same direction. Next, the alignment was computed using parameters for clustering closely related sequences and refined using realignment parameters optimized for distant organisms. Finally, tree construction was performed using Phylogenetic Analysis Using Parsimony (PAUP) or RAxML for Neigbour Joining (NJ) and maximum likelihood approaches, respectively. SGIV/GIV fall into a distinct cluster from the rest of the ranaviruses, a large group of viruses that infect fish, reptiles, and amphibians. Six megalocytiviruses formed a single well-separated clade. Furthermore, three fully-sequenced lymphocystivirus isolates appear to be more closely related to the two IIV genera than other vertebrate iridoviruses (VIVs). However, LCDV-1 was quite distant from LCDV-C/LCDV-Sa9. Based on this information, there is clear incongruence of the phylogenetic status of LCDVs with previous phylogenies constructed based on gene subsets. Our analysis also suggests the presence of two clades in *Chloriridovirus* including IIV-22, IIV-22a, IIV-25, IIV-30, and IIV-9/IIV-3. The AMIV virus [51] has not been classified yet; however, this analysis indicates that it possibly represents a new genus for IIVs. SHIV, a new iridovirid isolate (unclassified), also showed relatedness to both IIV genera (*Iridovirus* and *Chloriridovirus*) (Figures S1 and S2). Recent phylogenetic studies of SHIV based on different subsets (27 or 16 proteins) of concatenated iridovirid protein sequences conserved with SHIV orthologs suggested that SHIV should be considered a member of the proposed new genus “Xiairidovirus” [52,65] which is supported by our analysis presented in this review (Figures S1 and S2). Phylogenetic analysis also indicates that there is considerable diversity among members of both genera of invertebrate iridescent viruses.

## 3. Morphology and Composition

**Figure 3:**
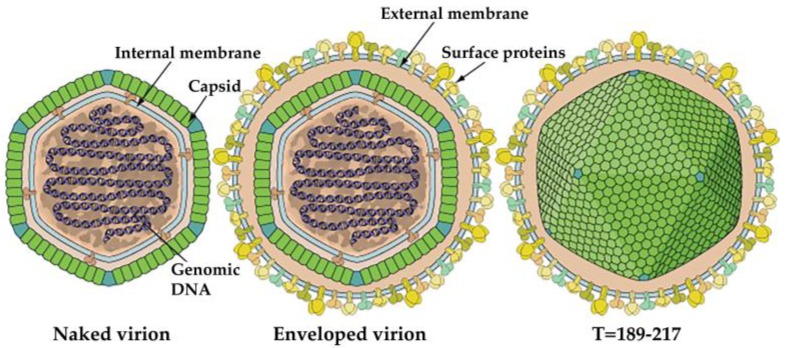
Graphical representation of the iridovirid structure [73]. Source: *ViralZone: www.expasy.org/viralzone, SIB Swiss Institute of Bioinformatics.*

As we mentioned earlier IIV-6 is the most studied member of IIVs with a large dsDNA genome packed into an icosahedral capsid with a T = 147 lattice. The virus particle is comprised of three concentric domains including the outer proteinaceous capsid layer, an intermediate lipid membrane with associated polypeptides and nucleocytoplasmic dsDNA genome (Figure 3) [3,66,67,68]. The size of IIV-6 virions was determined to be between 120-130nm in ultrathin sections. However, using CryoEM and 3D image reconstruction analyses on naked virions, [66] showed that IIV-6 can reach a maximum diameter of 185 nm when fibrils emanating from the surface of the virion were included in these measurements [66,67].

The lipid membrane of IIV-6 is comprised predominantly of phosphatidylinositol and diglycerides, which differs from the composition of the lipid membrane of its host cells, indicating that the viral lipid membrane is not likely derived from the host cell membrane [69]. Specifically, the major constituent of the viral lipid membrane is glycophosphatidylinositol (GPI), which acts as a lipid anchor to bind proteins via their C-terminus. The GPI-anchored proteins function as extracellular receptors or cell surface antigens/cell adhesion molecules, but their most important role is to provide a stable anchoring for extracellular proteases and lipases [70]. Other lipid membranes of invertebrate iridescent viruses (IIV-1, 2, 9, 21, 22, 30, 31, Anticarsia IV) showed similar lipid compositions to IIV-6 [71], suggesting that high GPI content might be a common feature of iridovirids. Since levels of glycerophospholipids are typically much higher in the endoplasmic reticulum (ER) than in other cellular compartments in mammalian and yeast cells [72], the ER may function as the cellular region where iridovirids gain their lipid components. Therefore, the ER of iridovirid-infected host cell should be analyzed.

## 4. IIV-6 Persistence and Sensitivity to External Factors

Some IIVs habitually can infect mosquitoes in aquatic habitats [74]. Owing to their structures, IIVs are highly stable in water as indicated by only small, 10-fold decreases in titres following 50 days either at 4 °C or at room temperature in aqueous suspensions [75]. To identify other environmental variables that influence virus stability, studies were performed investigating correlations between soil moisture levels and microorganisms on the stability of IIV-6. Although the virus was active across a range of soil moisture levels, dry soil rapidly inactivated the infectivity of IIV-6. Additionally, the half-life of IIV-6 propagated in non-sterile and sterile soil was recorded as 4.9 and 6.3 days, respectively, when compared to a control virus suspension (12.9 days) [76], indicating that extracellular enzymes or other environmental conditions influenced by the presence of microbes can impact the durability of IIV-6 in soil. However, the mechanisms by which microorganisms contribute to iridoviral persistence are not yet known.

IIV-6 is thermolabile and can be rapidly inactivated when the temperature is above 55 °C [77]. For example, IIV-6 viral activity decreased by approximately four logs when the virus was heated at 70 °C for 60 min or 80 °C for 30 min [78]. Furthermore, both solar UV light and ultraviolet radiation also reduced IIV-6 infectivity especially in aqueous habitats. Solar UV light caused a complete loss of infectivity (99.99%) after 36 h of exposure [79].

Exposure to various solvents and enzymes can also impact IIV-6 infectivity and sensitivity both in cell culture (i.e., *Spodoptera frugiperda* (Sf9) cells) and in whole insects (i.e., *Galleria mellonella* larvae) [80]. In addition, differences in sensitivity to the suspensions were observed depending on whether the assay was performed in cell culture or in whole insects. For example, while iridoviruses from both vertebrates and invertebrates were sensitive to ether, chloroform, sodium deoxycholate, only VIVs were sensitive to phospholipase A2 (PLA2) and only IIV-6 was sensitive to 1% Triton-x-100, 70% ethanol, and methanol (both in cell culture and in whole insects). Furthermore, no differences were detected in the infectivity of IIV-6 when the virus was treated with Tween-80, lipase, proteinase K, trypsin, magnesium chloride (MgCl_2_), ethylenediaminetetraacetic acid (EDTA) and other proteinases either in insect cell culture or whole insects. Notably, when the IIV-6 lipid bilayer was disrupted, the virus was still capable of infecting the cultured cells, but not insect larvae when the inoculum was injected into the haemocoel [78]. These observations highlight the need for studies on the role of the virus lipid component in the process of entry into invertebrate cells as well as host specificity and may also explain the observed differences in sensitivity to organic solvents and enzymes documented between vertebrate and invertebrate viruses. The roles of the lipid component in invertebrate IVs have not yet been reported; however, their role is well-documented in vertebrate IVs [81].

## 5. Host Range and Pathology

In the class Insecta, the host range of IIV-6 includes more than 100 species belonging to six orders; Coleoptera, Diptera, Hemiptera, Hymenoptera, Lepidoptera and Orthoptera, the natural hosts were reviewed by T. Williams [82]. In addition, several reports suggested that IIV-31 can naturally infect several different isopod species (Crustacea) [83,84,85] as well as *Drosophilidae* (Diptera) [86]. Iridovirid-like particles similar to IIVs have also been detected in marine invertebrates such as *Nautilus* spp. [87], the marine worm *Nereis diversicolor* [88] and from shrimp hemocytes [52,65] although the limited availability of genomic information or related viruses has hampered classification of these viruses.

Blue iridescent coloring is typically associated with patent infections, which occur due to the presence of crystalline arrays of particles in the cells of almost all tissues, especially the fat body [89]. This iridescent coloring of heavily infected invertebrates is the most obvious sign of IIV disease [68]. Patent IIV infections are almost consistently lethal; however, covert (inapparent) infections are more common, which may reduce the reproductive capacity and longevity of infected adult insects and arthropods [68,74,89,90,91,92,93,94,95]. In some cases, the link between viral presence and mortality is obscure. For example, Bromenshenk et al. [96] used mass-spectrometry-based proteomics to suggest a link between co-infection of *Nosema* species and IIV-6 with insect mortality; however, two subsequent studies failed to find a significant correlation between IIV-6 and colony collapse disorder (CCD) in the USA [97,98].

In addition to their natural hosts, IIV-6 can be propagated in twelve different insect cell lines [80], including *Drosophila* line 2 (DL2), *Drosophila* line 1 (DR1), *Aedes albopictus*, *Aedes aegypti*, *Anticarsia gemmatalis* (BCIRL-AG-AM), *Helicoverpa zea* (BCIRL-Hz-AM1), *Heliothis virescens* (BCIRL-HV-AM1), *Pieris rapae* (PR-5), *Plutella xylostella* (BCIRL-PX2-HNV3), *Spodoptera frugiperda* line 21 (Sf21), *Spodoptera frugiperda* line 9 (Sf9) and *Trichoplusia ni* (TN-CL1). Although all these cell lines are susceptible to infection, assays have indicated that the two *Drosophila* lines, DL2 and DR1, had the highest susceptibility to IIV-6 whereas cell lines from *Aedes albopictus* and *Plutella xylostella* were four orders of magnitude less susceptible compared to the others. Similar studies performed on cell lines derived from a hemipteran whitefly *Bemisia tabaci* [99], a leafhopper *Circulifer tenellus*, a lacewing, *Ceraeochrysa cubana* [100], a root weevil *Diaprepes abbreviatus* [89] and boll weevil *Anthonomus grandis* [101] showed that these cell lines were also susceptible to IIV-6 infection. Although it was reported that, IIV-6 can infect European corn borer *Ostrinia nubilalis* (Lepidoptera) larvae in several tissues and cells, including the fat body and hemocytes, a cell line derived from hemocytes of *Ostrinia nubilalis* could not be productively infected with IIV-6 [102], indicating that in some insects, extracellular host factors may influence viral infectivity and that not all cell lines derived from susceptible hosts can be infected with IIV-6.

Interestingly, reptiles and amphibians fed IIV-infected insects appear to become infected [103,104,105,106,107] and invertebrate iridovirus (IIV-6) propagation has also been achieved in some poikilothermic vertebrate cell lines e.g., a viper spleen cell line [108]. Furthermore, intraperitoneal injections of large doses of native or ultraviolet-irradiated IIV-6 were lethal to frogs and mice, while heat- or antiserum-inactivated IIV-6 had no lethal toxicity [109,110]. Although vertebrates can become infected with IIVs and lethality was observed under laboratory conditions and at high doses, natural infections are rarely symptomatic or lethal, suggesting that IIV replication is more limited in vertebrate hosts and that the host immune system is able to inhibit viral replication. Supporting the hypothesis of limited replication in vertebrates, IIVs tested in reptilian (VH2) and insect cell lines (Sf9) showed cytopathogenic effects similar to those of other reported iridoviruses [83], but viral titres were 2 logs higher in Sf9 compared to VH2. IIVs have been isolated from various stocks of crickets, suggesting that mass produced feeder insects could serve as a source of viral diseases for reptiles and amphibians kept as pets [103,111,112,113,114]. However, large-scale IIV outbreaks from ingestion of infected feeder insects have not yet been observed in reptiles or amphibians.

IIV-6 infection causes different immune responses in vertebrate and invertebrate hosts. Double stranded RNAs (ds-RNA) are the main activator of the innate immune system in insects and are produced by numerous RNA and DNA viruses during infection. dsRNAs are converted into viral small interfering RNAs (vsiRNA) by *Dicer* (*Dcr-2*) and then integrated with Argonaute-2 (AGO2) in the RNA-induced silencing complex (RISC) to facilitate the cleavage of the viral target RNAs [115]. Confirming the involvement of RNAi in IIV-6 immune responses, previous studies reported *Drosophila melanogaster Dcr2* or *AGO2* mutants were more sensitive to IIV-6 infection compared to wild type flies, which ultimately led to slight increases in viral titres [116]. Later, Kemp et al. [117] showed more dramatic increases in IIV-6 replication when different mutants of *Dcr2* and *AGO2* were used. Similar increases in viral replication were also observed in *rd2d* mutants. Furthermore, a subsequent study confirmed that the viral protein 340R suppressed RNAi responses in *D. melanogaster*, indicating that the virus encodes proteins that can interfere with insect RNAi immune responses. This viral protein binds to long dsRNAs and impedes the formation of vsiRNAs via Dicer-2 [115].

The contribution of the JAK-STAT pathway in antiviral immunity in *Drosophila* was tested by using loss-of-function mutants of the JAK kinase Hopscotch with IIV-6 and several RNA viruses [117]. Although the JAK-STAT pathway in *Drosophila* confers protection against some RNA viruses (e.g., DCV and CrPV, but not FHV, SINV, and VSV; [118], it does not provide immunity against IIV-6 infection [117]. In addition, epigenetic modifications associated with regulation of the JAK-STAT pathway could not be linked to IIV-6 viral responses in *Drosophila.* For example, H3 lysine 9 methyltransferase G9a, which is the one of the three H3K9 methyltransferases coded by *Drosophila* [119], regulates the JAK-STAT pathway and is required to regulate resistance to RNA viruses (e.g., nodaviruses) except DCV that shows a tolerance phenotype, but not to DNA viruses (e.g., IIV-6) [120]. Another study sought to address the involvement phagocytosis and autophagy in the control of viral infections in insects using a panel of six viruses including IIV-6. However, hemocytes did not respond to IIV-6 infection in *Drosophila* and infection did not trigger autophagy or apoptosis in virus-infected hemocytes, suggesting that autophagy and/or apoptosis may not be typical host responses to IIV-6 in *Drosophila* [121].

Vertebrates respond to IIV-6 using different immune responses from those of insects. Even though IIV-6 is a DNA virus, it can induce the type I IFN-dependent antiviral response in mammalian cells and is likely triggered by a cytosolic sensor, Retinoic acid inducible gene-I (RIG-I), upon viral dsRNA recognition. Transcription of viral DNA into RNA by RNA Polymerase III is required to activate the RLR pathway to obtain maximum IFN secretion [122]. In addition, activation of RLR-driven innate immune response against IIV-6 protects cells (Primary wild-type mouse embryonic fibroblasts) from possible infection with other viruses, such as Vesicular Stomattis and Kunjin arboviruses [122].

Overall, studies investigating the interactions between IIVs and invertebrate and vertebrate hosts suggest that IIVs can occasionally cross infect certain vertebrates, but that their immune systems may be able to inhibit replication under natural conditions, reducing or preventing symptoms.

## 6. Genomic Organization and Codon Usage

**Figure 4:**
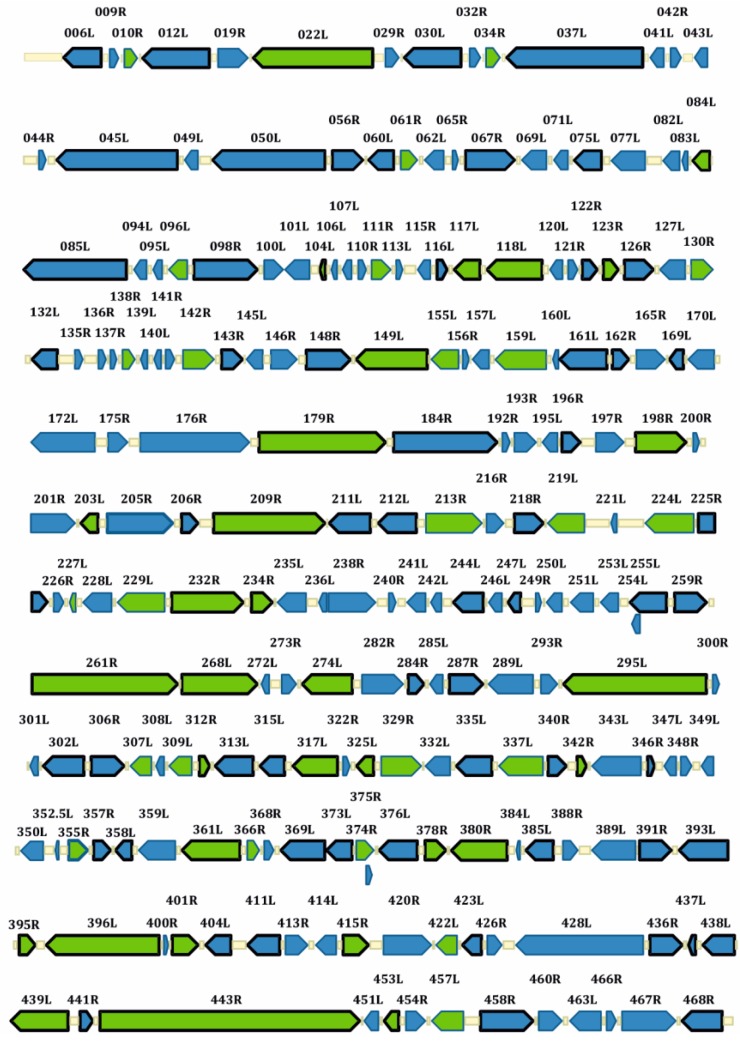
IIV-6 linear genomic map presenting virion and viral proteins identified in infected cells using mass spectrometry (genome size 212,482 bp). Arrows represent the transcriptional direction of the ORFs. All arrows with a black frame represent ORFs for which the corresponding protein was detected in the infected-cell proteomic analysis [127]. Green arrows represent known virion proteins [128]. This figure was published previously [127].

**Figure 5:**
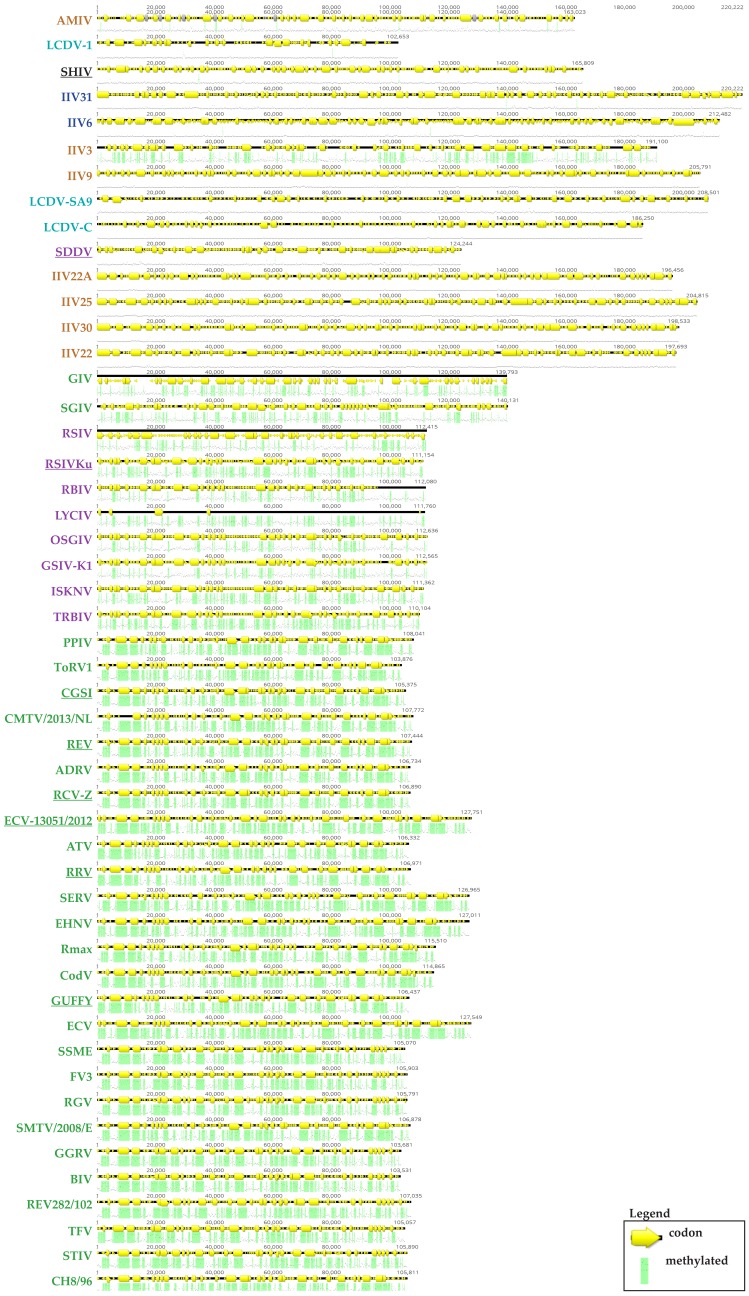
In silico prediction of methylation sites of 50 iridovirid genomes. The Geneious CpG Plugin (Biomatters Ltd., Auckland, New Zealand) predicts likely methylation sites according to the Hidden Markov models described by Richard Durbin. Green indicates potential methylation sites and yellow arrows indicates viral coding region of a genes. Methylation patterns IIVs versus VIVs could be distinguished, with the exceptions of IIV3 from IIVs and SDDV from SDDV as well as LCDV-1.

The IIV-6 genome consists of a single linear 212,482 bp dsDNA molecule with 28.63% G + C content [7]. Relative to other animal viruses, the IIV-6 genome has a unique arrangement with circular permutations and terminal redundancies, which causes variations in the number of direct terminal repeats during DNA replication and genome packaging [123,124]. While the coding capacity of the genome was originally estimated at 215 non-overlapping open reading frames (ORFs), the total number of ORFs rises to 468 when both non-overlapping and overlapping ORFs are considered (Figure 4). The revised coding boundaries of the 215 non-overlapping proteins are listed in the Uniprot database [53,54,55]. Importantly, gene transcripts of iridovirids have no evidence of introns [36,53,54] and viral mRNAs lack poly(A) tails [125]. However, biochemical and in silico evidence suggest the existence of viral microRNAs (miRNA) that may modulate viral gene expression [116]. In addition, important differences in methylation patterns have been observed in iridoviruses that infect invertebrates and vertebrates. For example, in contrast to iridoviruses that infect vertebrates (*Ranavirus*, *Lymphocystivirus* and *Megalovirus*), IIVs do not have a high frequency of methylation in their genomes, which is generally directed by viral DNA methyltransferases [126]. IIVs do not encode a DNA methyltransferase; however, VIVs have high levels of 5′-methylcytosine and code for a DMTase, except for SGIV [36]. We have performed in silico prediction of methylation sites of 50 iridovirid genomes and have demonstrated that there is obvious distinction of methylation patterns between IIVs and VIVs, except for IIV3 from IIVs and SDDV and LCDV-1 from VIVs that have yet unclear phylogenetic status. IIV3 showed predicted methylation sites in its genome while LCDV-1 had no obvious methylation sites predicted (Figure 5).

To date, 26 core genes have been identified in both vertebrate and invertebrate viruses within the family *Iridoviridae*, which have been linked to virus replication, gene transcription, protein structure and nucleocapsid assembly. The rest of the encoded proteins may have roles in virus-host interactions, hijacking of host cell machinery and establishment of viral infection. For example, core genes, including 022L, 037L, 142R, 143R, 176R/343L, 184R, 282R, 349L, 355R, 369L, 376L, 428L, and 436L, have key roles in viral transcription/DNA replication while 098R, 179R/439L and 380R have roles in protein processing, such as removal of signal sequences and modifications [129].

The core genes, 022L (nucleoside-triphosphatase) and 376L (ribonucleoside-diphosphate reductase) have roles in nucleic acid metabolism in the host cell. Other genes such as 037L (DNA polymerase), 142R (ribonuclease), 143R (putative protein), 184R (putative protein), 282R (putative protein), 176R/343L, 349L (transcription elongation factor), 428L (DNA-dependent RNA polymerase), 436L (signal localization protein), 355R (transcription regulator protein), and 369L (endonuclease homolog) are required for viral transcription/DNA replication while 098R (Serine-threonine protein kinase), 179R/439L (putative tyrosine kinase/lipopolysaccharide modifying enzyme) and 380R (Serine-threonine protein kinase), are required for protein processing such as removal of signal sequences and modifications [7,53,130]. The remaining ORFs related to core genes are identified as 274R (major capsid protein), 393L (immediate early protein), 75L (ATPase-like protein), 118L/458R and 337L (myristoylated membrane protein), 307L (a hypothetical protein of *Clostridium tetani*), 067R (helicase family protein), and 347L (ErvI/AIr family protein) whereas the functions of two core genes 295L and 287R are not yet clear [53].

## 7. Virion Proteins

IIV virion proteins have been previously analyzed in IIV-1 [131] (Watson and Seligy, 1997), IIV-6 [128,132,133] and IIV-9 [13]. Additionally, one- or two-dimensional SDS-PAGE methods were carried out to characterize the polypeptides in IIV-6 virions, which revealed 21–28 polypeptides and 35 polypeptides, respectively [132,133,134]. An additional study performed by Kelly and Tinsley [134] detected 20 polypeptides from IIV-2. Elliot et al. [135] identified and compared the polypeptides from IIV-2, IIV-22 and IIV-25 and demonstrated that IIV-22 and IIV-25 have 25 polypeptides that differ markedly in terms of molecular weight from the 20 polypeptides identified from IIV-2 [134]. However, in these earlier studies, we have observed that different experimental settings can affect the resolution of the gel analyses and could have thus influenced previous results and comparisons.

More recently, a comprehensive proteomic analysis using 1- and 2-dimensional SDS-PAGE combined with LC-MS/MS was performed to identify IIV-6 virion proteins and 54 proteins were detected [128]. Possible functions of fifteen of the putative IIV-6 ORFs were inferred by blast searches and included serine-threonine kinase, 209R, 380R, 439R; dual specificity protein phosphatase, 123R; DNA polymerase (viral) N terminal domain, 232R; carboxy terminal domain phosphatase, 355R; nucleodide triphophatase (NTP) I, 22L; fasciclin, 96L; ribonuclease III, 142R; tyrosine protein kinase, 179R; cathepsin, 361L; DNA-binding protein, 401R; protein disulfide isomerase, 453L; lysosome associated membrane glycoprotein, 061R and a ranavirus enveloped protein homolog, 118L. The remaining 39 proteins lacked similarity to any other annotated viral proteins and their functions should be investigated further. In addition, the molecular masses of the finger and zip proteins, the anchor protein, and the monomer of the pentameric complex were estimated as 19.7, 11.9, 32.4 and 39.3 kDa, respectively [67]. Based on these size approximations, seven candidate genes coding for zip proteins were identified, including; 010R, 138R and 321R, which had estimated sizes ranging from 10.5 to 13.3 kDa. The predicted size of the protein products of ORFs 329R and 219L were similar to the monomer of the pentameric complex and 457L and 142R represent candidates for anchor proteins [128]. Genomic analysis of Wiseana iridescent virus (IIV-9) identified 191 predicted genes, with 20% of its repeated sequences mainly located within the coding regions. 97 of 211 IIV-6 genes have detectable orthologs in IIV-9, whereas 108 out of 191 IIV-9 genes have orthologs in IIV-3. Additionally, proteomic analysis of IIV-9 revealed 64 virion proteins and, when SF21 cells were infected by IIV-9, the presence of 94 viral proteins in infected cells were confirmed [13].

Phosphorylation reactions are important post-translational protein modifications that can modify or regulate the function of a protein. They also represent common host responses to iridovirid infection. During IIV-6 infection, increased phosphorylation of the ribosome associated proteins during the early phase of the infection cycle requires the expression of the viral genome [136]. However, phosphorylation events were observed in ribosomal proteins when permissive cells were infected with UV-irradiated IIV-6, suggesting that increasing phosphorylation reactions were likely a host cell response rather than being related to an expression of the viral genome.

Iridovirid genomes contain multiple genes coding for kinases and phosphatases that perform important post-translational modifications of proteins to help regulate protein activities over the course of the infection [127]. Active expression of these virus-encoded phosphatases and kinases involved in dephosphorylation and phosphorylation reactions was observed by proteomic analysis of infected cells. Four protein kinases, three putative serine/threonine kinases (ORFs 209R, 380R and 439R) and one tyrosine protein kinase (179R) were identified from the proteome of IIV-6 virions, while an additional protein kinase (098R) was identified from the viral proteome of infected cells. Two phosphatases were identified from virions, which included a dual specificity protein phosphatase, 123R and carboxy terminal domain phosphatase, 355R.

Although kinases and phosphatases are among the most abundant proteins, it is challenging to assign functions to these enzymes without expressing them individually in cell culture, making knock-out viruses, unravelling their subcellular localization, or identifying the protein targets that are specifically phosphorylated. However, the roles of several kinases have been elucidated. One putative serine/threonine kinase (380R) was present in high abundances in IIV-6 virions [127]. A purified serine/threonine protein kinase 389L (also called iridoptin) induced apoptosis in insect cells and was initially thought to be associated with the IIV-6 virion structure [137]. However, no peptides were identified from this protein in either the virion proteome or the proteome from infected cells [127,128]. Thus, it remains uncertain whether 389L is a virion associated protein or not.

## 8. Viral Entry, Replication, and Release Strategy

**Figure 6:**
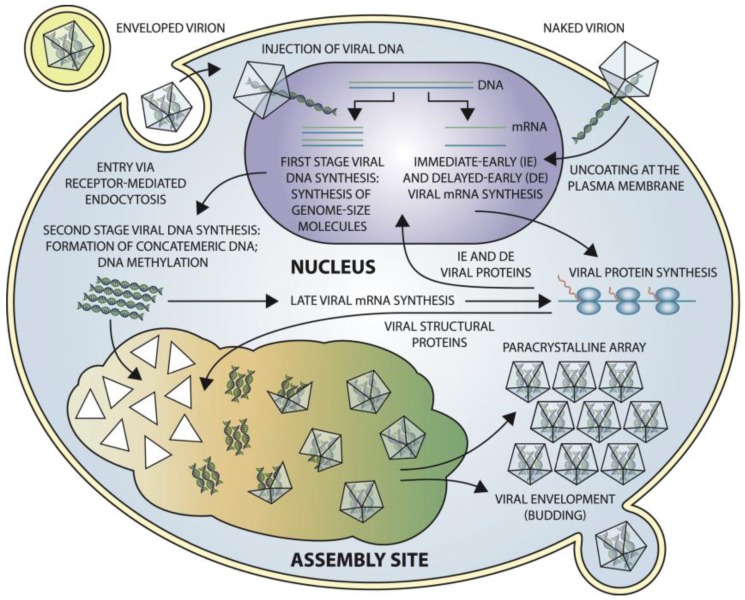
Schematic representation of iridoviral replication. This figure was obtained from the 10th report of ICTV with permission [1,2].

Frog virus 3 (FV3) is the best-characterized member of the genus (*Ranavirus*) and the family (*Iridoviridae*) and has served as a model to elucidate iridoviral transcription, genome replication and virus-mediated host-shutoff [138] (Figure 6). Iridovirids have nucleocytoplasmic replication. Initially, the virus attaches to cell surface receptors, and the virus is engulfed by the host cell via clathrin-mediated endocytosis or macropinocytosis in a pH-dependent manner [139]. ORF 096L of IIV-6, annotated as a fasciclin domain, is proposed to act as an insect cell adhesion molecule (CAM) [140] which may facilitate “fusion” between the virus shell and the cellular membranes [141].

Following the entry into the host cell, immediate early and delayed early transcripts are synthesized using the virion DNA as a template [142,143,144]. These transcripts encode proteins that are crucial for DNA replication and expression of late genes [133]. Then, newly synthesized viral DNA is translocated from the nucleus to the cytoplasm where a second stage of viral DNA replication occurs by the formation of DNA concatemers [145] (Figure 6). Late viral transcripts, including transcripts of the structural proteins for virion formation, are likely synthesized by a host RNA polymerase or a viral encoded RNA polymerase that has not yet been identified in IIVs. Evidence from VIVs seems to suggest that late viral messages are synthesized by a virus-encoded transcriptase. This hypothesis is supported because knocking down the large subunit of the viral transcriptase of SGIV with asMOs (antisense morpholino oligonucleotides) eliminates late protein synthesis [146]. Once the replicated DNA has reached the cytoplasm, it forms large concatemers, which are eventually packaged into virions. Progeny virions accumulate in large paracrystalline arrays or egress from the cell by either budding or cell lysis, as mentioned previously (Figure 6).

According to the mechanism by which viral particles are released from host cells, viruses can be either be characterized as “extracellular viruses”, which are released via budding from the host cell membrane or “intracellular (naked) viruses”, which are released along with mature virions from infected cells via lysis (Figure 6). The mechanism of release from host cells is unknown for IIVs. However, the virion assembly and budding process have recently been illustrated for a vertebrate iridovirus (SGIV) using a high pressure freezing (HPR) method. The mitochondria surrounding the viral assembly site (VAS) became enlarged and eventually lost their inner cristae, suggesting that they may eventually become empty vesicles, vulnerable to viral invasion. Mature virions can bud into those vesicles and these virus-filled vesicles may fuse with one another to form larger vacuoles. Eventually, cell lysis will release the mature virions from the cell. This new budding method may be an immune system evasion strategy of SGIV [147].

The specific classes of lipids in the virion structure can influence virus infectivity [148]. In addition, changes in host cellular lipid composition associated with cell proliferation, apoptosis, cellular stress and viral infection can also occur during viral replication and may also influence virus-host interactions [81] and impact the virus’ ability to infect its hosts. For example, infection with IIV-6 can induce modifications in the phospholipids of invertebrate cells by decreasing the incorporation of phosphorous into cell phospholipids and stimulating phosphatidylcholine synthesis [149]. To understand viral lipid–protein interactions, entry process, intracellular trafficking, viral assembly and releasing strategy, the lipid composition of the vertebrate iridovirus SGIV was quantitatively analyzed using a combined approach of liquid chromatography and mass spectrometry. The study revealed that selective losses of some of the lipid classes can significantly diminish viral infectivity. For example, enzymatic digestion of viral lipids with phospholipases and sphingomyelinase decreased viral infectivity [81], but did not otherwise compromise the integrity of the viral capsid proteins. As expected, recovery of the viral lipids with liposomes obtained from a grouper embryonic cell line (GEC) restored the infectivity of the virus [147]. In addition, as discussed earlier, disruption of the lipid bilayer in IIVs had a profound effect on their ability to develop in vivo infections [78].

## 9. Transcriptional Regulation

In IIV-6 infected cells, viral transcription occurs, in a regulated temporal gene expression cascade that can be subdivided into three temporal classes: immediate-early (IE or α), delayed-early (DE, β), and late (L, γ), with the majority of the genes expressed as IE genes [125,142,143,144]. Both *cis*-acting and *trans*-acting regulatory factors initiate the transcription that coincide with various stages of viral infection. However, some IE and DE transcripts are still present during the late phase of infection and no meaningful correlation has yet been identified between transcription and the prevalence of particular proteins during infection [127,143]. Overall, a previous study performed by northern blot analyses using putative gene-specific probes on total cellular RNA from IIV-6 infected IPRI-CF-124T cells confirmed the expression of 137 viral transcripts including, 38 IE, 34 DE and 65 L gene transcripts. Proteins associated with virions are likely key activators of immediate early genes. For example, purified IIV-6 DNA is inadequate to achieve infection; however, infectivity can be recovered by adding virion proteins, suggesting that these proteins are required to trigger transcription of early genes [150,151]. Furthermore, IE class transcripts appeared around 0.5 h post infection (h.p.i) while the transcription of the delayed-early class genes was detected around 3 h.p.i. The expression of DE genes was impeded when protein synthesis inhibitors were applied which clearly indicates that DE genes require at least one earlier gene product to initiate transcription. In the late class genes, 65 transcripts were detected around at 6 h.p.i. [143].

A more recent study provided detailed information about the kinetics of IIV-6 viral protein levels in infected *Drosophila* S2 cells using a label-free quantitative proteomic approach. The transcripts belonging to the IE class included 022L, 104L, 118L, 123L, 155L, 179R, 209R, 232R, 261R, 268L, 295L, 312R, 361L, 380R, 415R, 439L, and 453L. These gene products are mainly involved in blocking host cell apoptosis, nucleoside metabolism, post-translational protein modifications and transcriptional activation of DE genes [125,143]. DE genes included 117L, 149L, 198R, 229L, 378R, 396L, 457L, which function as viral DNA polymerases, protein kinases, and transcriptional activators of late genes. The major capsid protein MCP (274L), one of the most abundant proteins in IIV-6, was expressed with other late class transcripts, including 247L, 234R, 317L, 342R and 401R [127].

## 10. Promoter Elements and Transcription Initiation Sites

It is difficult to conclusively identify promoter elements that regulate the temporal expression patterns of iridovirid genes [53]. However, several studies have led to the identification of motifs in putative promoter regions that may be important for transcriptional regulation. For example, in IIV-6, the transcription profiles and promoter structures of the DNA polymerase (*DNApol*; 037L) and major capsid protein genes (*mcp*; 274L) have been analyzed in detail [152]. IIV-6 infection of *Bombyx mori* SPC-BM-36 cells with using DNA or protein synthesis inhibitors demonstrated that *DNApol* belongs to the IE gene class whereas *mcp* is transcribed as a late gene. An AAAAT motif located between the −19 and −2 region was determined to be responsible for the promoter activity of *DNApol*. 

A second IE promotor study clearly showed that sequences between −20 and −10 relative to the transcription start site have key promoter activity for IIV-6 012L. Although similar motifs were not found in the upstream regions of any other potential IIV-6 IE genes, the promoter regions of IE and DE genes appear to differ in their organization in IIV-6. The major differences in promoter organization were in the 20 nt region upstream of the transcription initiation site [151,153]. IIV-6 virion proteins are likely able to either directly or indirectly activate the promoters to trigger transcription of some IE genes. Supporting this hypothesis, reporter assays confirmed that without virion proteins the promoters were inactive even though the reporter plasmid constructs carrying these promoters were transfected.

Interestingly, *DNApol*, *helicase*, and *mcp* transcripts of IIV-6 lack polyA tails which was discovered when oligo-DT primers in reverse transcriptase reactions did not lead to any amplification. Furthermore, clear polyadenylation signals downstream of these two IIV-6 ORFs were not found [152]. To identify the transcription termination signals of IIV-6 genes, the LACE (Ligation-based amplification of cDNA ends) method was implemented to amplify the 3′ UTR regions of viral transcripts [125]. LACE showed that about half of all IIV-6 genes contained complementary TAATG and CATTA motifs in the 3′ regions of their mRNAs. These CATTA motifs may be conserved features that enable the formation of hairpins (22–56 nt in length) in IIVs as they are missing in many vertebrate viruses. In the absence of polyadenylation signals, these hairpins may serve as transcriptional terminators [154].

## 11. Induction/Inhibition of Apoptosis in Infections

Although the signaling pathways and recruited proteins linked to apoptosis can vary between vertebrates and invertebrates, the principles and the dynamics of apoptosis in response to iridovirid infection are similar. During the early stages of iridovirid infection, both vertebrate and invertebrate host cells trigger apoptotic machinery to impede the cell-to-cell movement of progeny viruses [155,156]; however, many viruses have evolved mechanisms to protect the progeny viruses from the adaptive immune system and enhance the likelihood of spreading to neighboring cells by producing anti-apoptotic proteins [157,158]. The expression of these anti-apoptotic genes prolongs survival of infected cells, which increases the production of progeny virus and/or may lead to virus persistence. Furthermore, viruses contain genes that may stimulate apoptosis (protein tyrosine phosphate or viral kinases), which may contribute to viral dissemination to neighboring cells. Fish iridoviruses, for instance, appear to exploit apoptosis for their dissemination.

Initially, expression of early viral gene/s (IE) inhibited apoptosis, which suggests that one or more IE genes likely functions as anti-apoptotic factors (e.g., *iap*). Among members of the genus *Iridovirus*, IIV-6 was the first virus identified to contain three ORFs (157L, 193R, and 332L) with homology to the *iap* (*inhibitor of apoptosis*) gene of a baculovirus [158]. Among these genes, only 193R, which was expressed as an IE gene, was confirmed as a functional IAP, [158]. The other two *iap* candidate ORFs, 157L and 332L, contained only RING finger domains and lacked the BIR domains, suggesting that they likely do not directly inhibit apoptosis. A subsequent study also identified a putative *iap* gene in a recently sequenced *Armadillidium vulgare* iridescent virus (IIV-31) [9] which shares homology with 193R of IIV-6 but its functionality has not yet been tested. IIVs also code for pro-apoptotic factors, which trigger apoptosis at later stages during the infection to facilitate viral dissemination. For example, IIV-6 infected SPC-Bm-36 cells formed apoptotic-like vesicles after 18 h of infection [159] and further evidence exists that pro-apoptotic factors may be associated with virions. For example, a comprehensive study showed that high doses of IIV-6 can initiate apoptotic responses, yet at lower doses, apoptosis was inhibited [156]. Further supporting the hypothesis of virion associated pro-apoptotic factors, de novo synthesis of viral proteins was not required for induction of apoptosis [156] and IIV-6 virion protein extract induced apoptosis in spruce budworm and boll weevil cell lines, as detected by blebbing, DNA fragmentation, TUNEL assay and protein kinase activity [160]. More recently, an *istk* gene product and a virion protein of IIV-6, 389L, which is a 49 kDa polypeptide, was identified as apoptosis inducing factor. Additionally, a 35-kDa cleavage product of ISTK (iridoptin) also functions as a potent inducer of apoptosis in host cells [137] however it is unknown when it is produced in infection and whether it is a virion or ICP protein [127,128].

## 12. Concluding Remarks

Recent genomic, transcriptomic, proteomic, and molecular studies have elucidated the routes of infection, mechanisms of replication, classification, and the identities of viral proteins that are associated with virulence or modulate host cellular responses to IIV infection. Despite these recent advances, several important knowledge gaps exist. For example, it is not known which host and/or viral factors trigger viral entry and replication into host cells and the regulatory mechanisms of genes expressed at various stages during infection remain uncharacterized. While transcriptomic and proteomic studies may address some of these short-comings, other important questions regarding iridovirid-host interactions cannot be addressed without the use of functional genomics. Previously, functional genomics studies in IIVs have been hampered by the lack of genetic recombination systems for producing genetic (knockout) mutants of the virus; however, the first recombinant IIV using a homologous recombination site was recently established [161,162]. The identification of viral proteins responsible for viral infectivity will be an important next step towards the development of a genetic recombination system for iridovirids (possibly similar to a bacmid system) as iridovirid DNA by itself is not infectious. The development of a recombination system will expand the utility of IIVs as a model for identifying and characterizing iridovirid genetic factors that drive virus-host interactions, impact host fitness, and influence immune system response, which could ultimately help limit or prevent disease in aquatic ecosystems or lead to the development of IIVs as biocontrol agents against medically important vectors.

Next, IIV-6 has attracted little attention as a potential biopesticide [163] due to its broad host range and limited mortality, although epizootics have been reported occasionally [82,90,164]. However, transcriptomics and proteomics studies have identified virally coded genes that may be linked to host toxicity and could be exploited as biocontrol compounds. For example, serine/threonine kinases can induce toxicity in several insect groups, such as weevils and whiteflies. Specifically, the serine/threonine kinase “iridoptin” has been identified as a potential viral toxin against non-lepidopteran insects, a group of insects for which there are limited options for biological control [137]. Additional functional genomics studies along with an expanding repertoire of genomic resources may lead to the identification of new virulence factors and toxins that could be exploited and engineered for targeted control against certain groups of insects, for example, insects of agricultural importance or that transmit plant pathogens and/or disease-causing parasites, such as mosquitoes, whiteflies and grasshoppers [89,100,113,165,166]. Furthermore, virally coded kinases could represent potential antiviral drug targets, as they play crucial roles to guarantee successful viral infection [130]. Understanding the roles of these kinases in IIV-6 may help to develop therapeutics against devastating iridovirus outbreaks in fish farming, for example.

Finally, current phylogenetic approaches for iridovirids have a limited ability to conclusively resolve species level differences; however, the availability of additional genomic resources could help to overcome some of the limitations of current phylogenetic methods and also improve support for proposed classifications. Specifically, we propose to establish a phylogenetic approach using raw sequencing data to incorporate pan-genomic information into the analysis of viral phylogenies, which may ultimately lead to the development of new classification criteria. This approach would allow researchers to leverage sequences of viral quasispecies in infected cells and/or host organisms [167], to extract neglected sequence data due to infectome complexity and gauge the genetic diversity and complexity of a viral species, e.g., gene variants, fusions and recombination products. Ultimately, pan-genomic information may expand the capacity to resolve phylogenetic relationships beyond the capabilities of current methods based on using gene subsets or complete genome information from one representative isolate.

## Figures and Tables

**Table 1:** Comparison of features of iridovirids.

Sub-Qualification	*Genus*	Virion Size	Hosts	GC Content	DNA Methylation
*Betairidovirinae*	*Iridovirus*	120–130 nm	arthropods, particularly insects	29–32%	absent
*Betairidovirinae*	*Chloriridovirus*	180 nm	Diptera with aquatic larval stages, mainly mosquitoes	48%	absent
*Alphairidovirinae*	*Ranavirus*	150 nm	Reptilia, Amphibia and Osteichthyes	54%	present
*Alphairidovirinae*	*Lymphocystivirus*	198–227 nm	flounder, plaice, and dab	29.1–33%	present
*Alphairidovirinae*	*Megalocytivirus*	140–200 nm	sea fish	54.8%	present

**Table 2:** Identified iridovirids with complete genome sequences. IIV-6—Invertebrate iridescent virus 6; IIV-31—Invertebrate iridescent virus 31; IIV-3—Invertebrate iridescent virus 3; IIV-9—Invertebrate iridescent virus 9; IIV-22—Invertebrate iridescent virus 22; IIV-22A—Invertebrate iridescent virus 22A; IIV-25—Invertebrate iridescent virus 25; IIV-30—Invertebrate iridescent virus 30; ADRV—Andrias davidianus ranavirus; ATV—Ambystoma tigrinum virus; BIV-ME—Bohle iridovirus isolate ME 93/35; CGSI-HN1104—Chinese giant salamander iridovirus isolate HN1104; CH8/96—Testudo hermanni ranavirus; CMTV/2008/E—Common midwife toad virus—2008/E; CMTV/2013/NL—Common midwife toad virus—2013/NL; CodV—Cod ranavirus; DFV—Doctor fish virus; ECV-13051/2012—European catfish virus; ECV-14612/12—European catfish virus; EHNV—Epizootic haematopoietic necrosis virus; ESV—European sheatfish virus; FV3—Frog virus; GGRV—German gecko ranavirus; GIV—Giant sea perch iridovirus; ATV-GUFFY—Ambystoma tigrinum virus isolate GUFFY; GV6—Guppy virus 6; LMBV—largemouth bass virus; PPIV—Pike-perch iridovirus; RCV-Z—Rana catesbeiana virus Z; REV 282/102—Rana esculenta virus isolate 282/102; RGV—Rana gryliovirus; RMax—Ranavirus maxima; RRV—Regina ranavirus; SERV—Short-finned eel ranavirus; SGIV—Singapore grouper iridovirus; SSME—Spotted salamander population in Maine; STIV—Soft-shelled turtle iridovirus; TFV—Tiger frog virus; ToRV-1—Tortoise ranavirus 1; LCDV-1—Lymphocystis disease virus 1; LCDV-C—Lymphocystis disease virus—China; LCDV-Sa—Lymphocystis disease virus—Sparus aurata; GSIV-K1—Giant sea perch iridovirus; ISKNV—Infectious spleen and kidney necrosis virus; LYCIV—Large yellow croaker iridovirus; OSGIV—Orange spotted grouper iridovirus; RBIV—Rock bream iridovirus; RSIV—Red seabream iridovirus; SDDV—Scale drop disease virus; TRBIV—Turbot reddish body iridovirus; AMIV—Anopheles minimus iridovirus; SHIV—shrimp hemocyte iridescent virus.

Genus (Subfamily)	Species, Strains, and Isolates	Size (bp)	ORFs	G + C %	GenBank Accession Number	References
*Iridovirus*	IIV-6	212,482	215 *	28.63	AF303741	[7]
	IIV-31	220,222	203	35.09	HF920637	[11]
*Chloriridovirus*	IIV-3	191,132	126	48	DQ643392	[12]
	IIV-9	205,791	191	31	GQ918152	[13]
	IIV-22	197,693	167	28.05	HF920633	[14]
	IIV-22A	196,456	174	28	HF920634	[15]
	IIV-25	204,815	177	30.32	HF920635	[16]
	IIV-30	198,533	177	28.1	HF920636	[17]
	IIV-3	191,132	126	48	DQ643392	[12]
*Ranavirus*	ADRV	106,734	101	55	KC865735	[18]
	ATV	106,332	96	54	AY150217	[19]
	BIV-ME	103,531	100	55.2	KX185156	[20]
	CGSI–HN1104	105,375	111	55.2	KF512820	Unpublished
	CH8/96	105,811	75	55	KP266741	[21]
	CMTV/2008/E	106,878	104	55.3	JQ231222	[22]
	CMTV/2013/NL	107,772	104	55.3	KP056312	[23]
	CodV	114,865	98	54.9	KX574342	[24]
	DFV	ND	ND	ND	ND	Unpublished
	ECV-13051/2012	127,751	135	54.2	KT989884	Unpublished
	ECV-14612/12	127,549	136	54	KT989885	Unpublished
	EHNV	127,011	100	54.05	NC028461	[25]
	ESV	127,732	136	54.23	JQ724856	[26]
	FV3	105,903	98	55	AY548484	[27]
	GGRV	103,681	73	55	KP266742	[21]
	GIV	139,793	120	49	AY666015	[28]
	ATV-GUFFY	106,437	99	54	KR075882	[29]
	GV6	ND	ND	ND	ND	Unpublished
	LMBV	ND	ND	ND	ND	Unpublished
	PPIV	108,041	109	55.3	KX574341	[30]
	RCV-Z	106,890	98	55	MF187210	[31]
	REV 282/102	107,444	101	55.2	MF538628	[32]
	RGV	105,791	106	55	JQ654586	[33]
	Rmax	115,510	100	54.9	KX574343	[24]
	ATV-RRV	106,971	102	54.1	KR075879	[34]
	SERV	126,965	111	54.7	KX353311	[35]
	SGIV	140,131	162	48.64	AY521625	[36]
	SSME	105,070	95	55	KJ175144	[37]
	STIV	105,890	105	55.1	EU627010	[38]
	TFV	105,057	105	55.01	AF389451	[39]
	ToRV-1	103,876	76	55	KP266743	[21]
*Lymphocystivirus*	LCDV-1	102,653	108	29.1	NC_00182	[40]
	LCDV-C	186,250	239	27.2	AY380826	[41]
	LCDV-Sa	208,501	183	33	KX643370	[42]
*Megalocytivirus*	GSIV-K1	112,565	135	53.02	KT804738	[43]
	ISKNV	111,362	125	54.8	AF371960	[44]
	LYCIV	111,760	ND	53.92	AY779031	[45]
	OSGIV	112,636	126	54	AY894343	[46]
	RBIV-KOR-TY1	112,080	116	53	AY532606	[47]
	RSIV	112,415	93	54	AB104413	[48]
	SDDV	124,244	129	37	KR139659	[49]
	TRBIV	110,104	115	55	GQ273492	[50]
Unclassified	AMIV	163,023	148	39	KF938901	[51]
	SHIV	165,809	170	34.6	MF599468	[52]

* The IIV-6 genome contains a total of 468 ORFs when both overlapping and non-overlapping protein coding ORFs are considered [7] and 215 ORFs when only non-overlapping coding regions are considered. The revised boundaries for the 215 non-overlapping proteins are listed in Uniprot database [53,54,55].

## Supplementary Materials

The following are available online at http://www.mdpi.com/1999-4915/10/4/161/s1, Figure S1: Neigbour-joining phylogeny of family *Iridoviridae*, Figure S2: RAxML Maximum likelihood phylogeny of family *Iridoviridae*.
