# Supplementary material for: Invertebrate Iridoviruses: A Glance over the Last Decade
Source: Viruses. 2018 Mar 30;10(4):161. doi: 10.3390/v10040161 (PMC5923455; doi:10.3390/v10040161)
Supplement: Supplementary file 1 [file viruses-10-00161-s001.pdf]

Supplementary Figure Legends

Figure S1. Neighbour-joining phylogeny of family *Iridoviridae.* Whole genome sequences were aligned with MUSCLE using diagonal optimization with 2 iterations. The distance measure for the first iteration kmer4_6 for closely related species and the distance for the second iteration was Neighbour-joining (clustering method) for more distantly related species. CLUSTALW was used for the sequence weighting scheme and the hydrophobicity multiplier was set to 1.2. The phylogenetic tree was constructed using PAUP implemented in Geneious with the Neighbour-joining method with 100 bootstrap pseudoreplicates, allowing outgroups and using a heuristic search. Nucleotide substitution model tests were performed using hLRT (hierarchical likelihood ratio test) with Tamura-Nei [168]. Jukes-Cantor [169], and HKY [170] were selected as candidates for NJ analysis. Bootstrap support values for 100 pseudoreplicates are displayed as percentages. Different models produced similar trees. Nodes that are missing bootstrap values have 100% support. Branches are coloured according to Chinchar *et al.* [1,2] (Figure 3) and are used to indicate different iridovirid genera. Newly added viruses are underlined [171,172].

Figure S2. RAxML Maximum likelihood phylogeny of family *Iridoviridae*. Complete iridovirid genome sequences were aligned as described in Figure S1. The distances were visualized by Genious R11 and the maximum likelihood tree was constructed by RAxML using the following parameters: GTR GAMMA: GTR+Optimization of substitution rates+GAMMA model rate of heterogeneity, -f a -x 1, and parsimony random seed 8. Rapid bootstrapping was performed using 100 bootstrap pseudoreplicates). Newly added viruses are underlined [173,174,172].

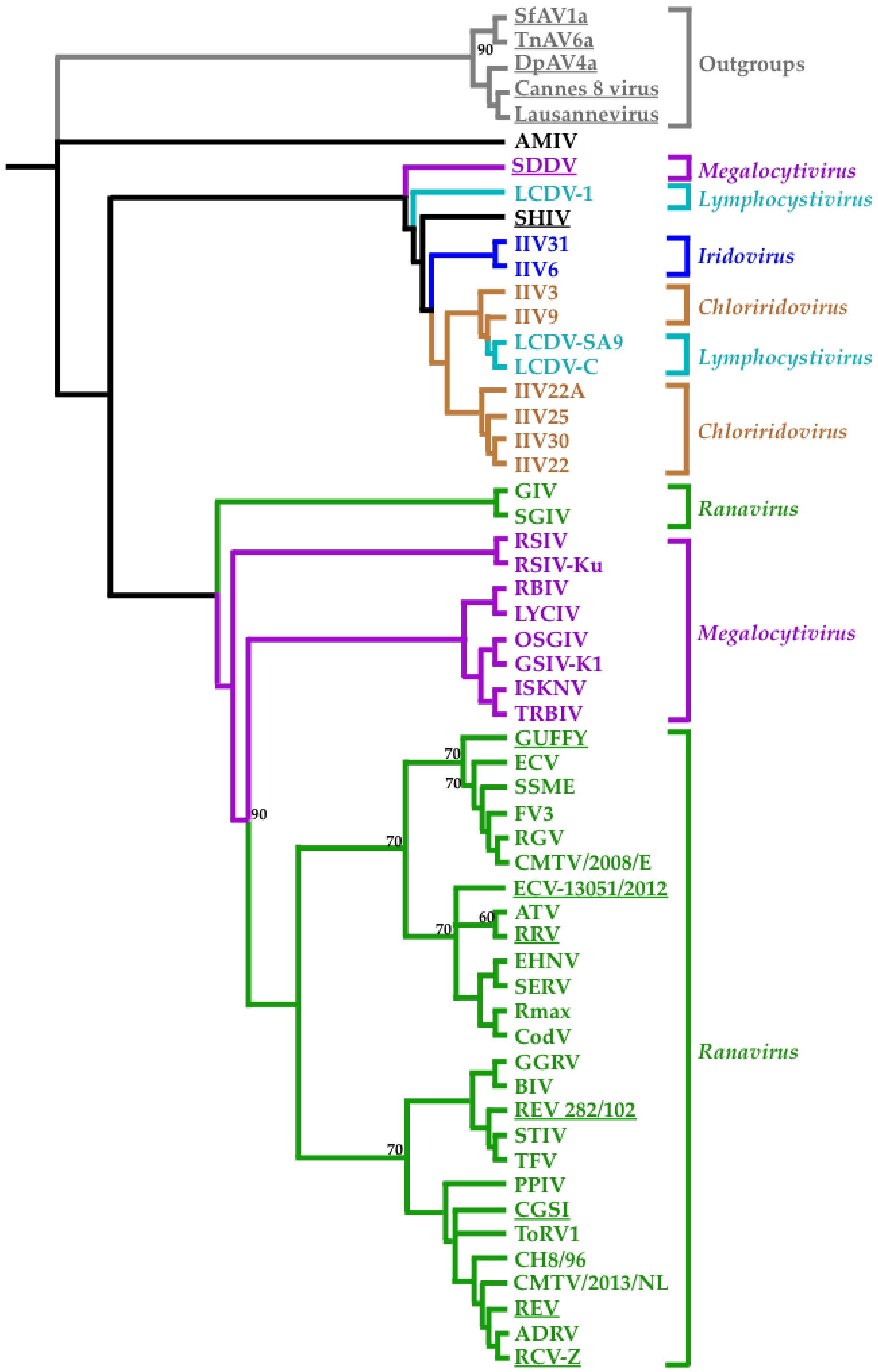

Figure S1. Neighbour-joining phylogeny of family *Iridoviridae*.

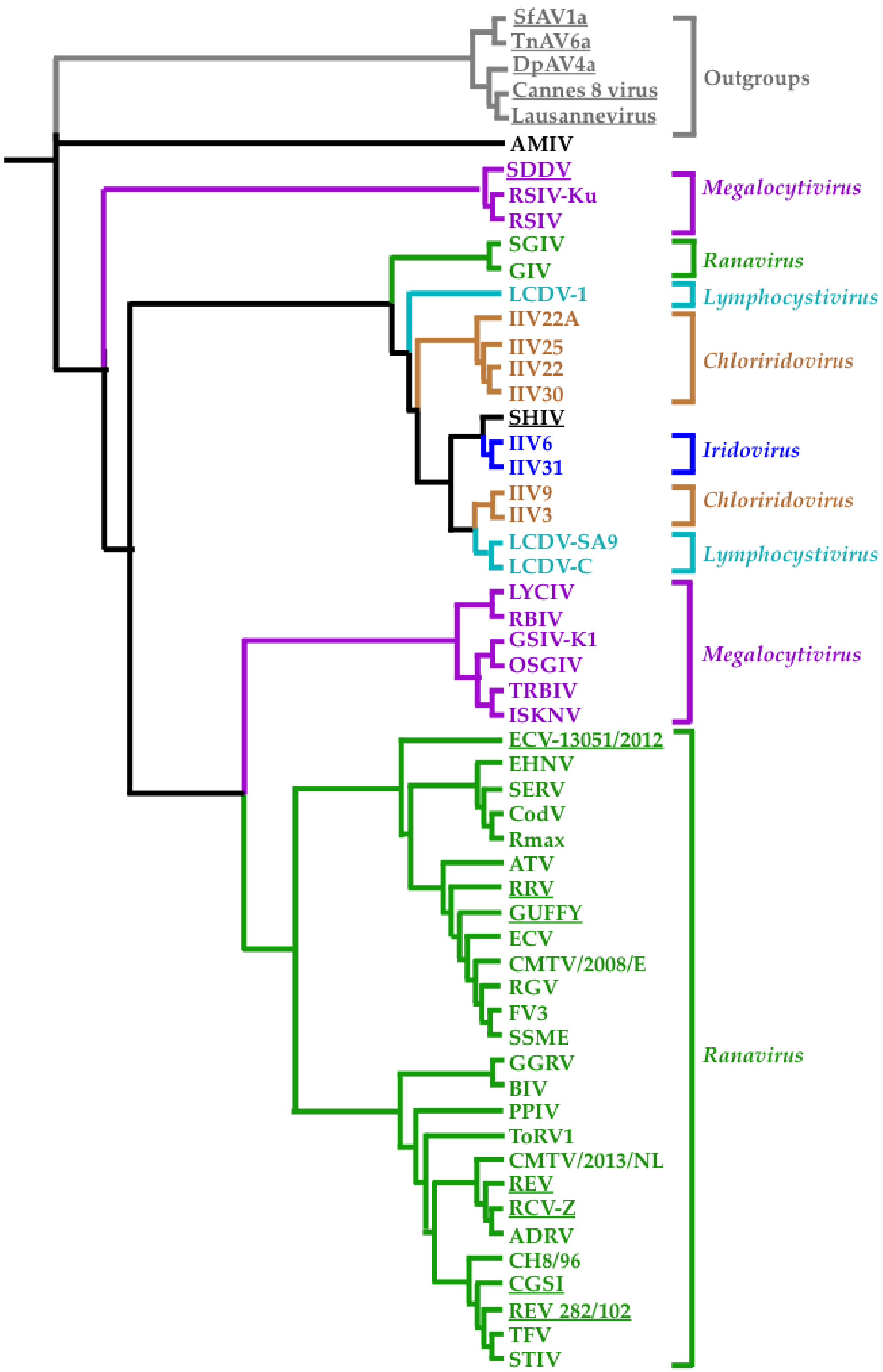

Figure S2. RAxML Maximum likelihood phylogeny of family *Iridoviridae*.

168. Tamura, K.; Nei, M. Estimation of the number of nucleotide substitutions in the control region of mitochondrial DNA in humans and chimpanzees. *Mol. Biol. Evol.* **1993**, *10*, 512–526.
169. Jukes, T.H.; Cantor, C.R. Evolution of protein molecules. In *Mammalian Protein Metabolism*; Academic Press: New York, NY, USA, 1969; pp. 21–132.
170. Hasegawa, M.; Kishino, K.; Yano, T. Dating the human-ape splitting by a molecular clock of mitochondrial DNA. *J. Mol. Evol.* **1985**, *22*, 160–174.
171. Wilgenbusch, J.C.; Warren, D.L.; Swofford, D.L. AWTY: A System for Graphical Exploration of MCMC Convergence in Bayesian Phylogenetic Inference. 2004. Available online: http://ceb.csit.fsu.edu/awty (accessed on 26 March 2018).
172. Kearse, M.; Moir, R.; Wilson, A.; Stones-Havas, S.; Cheung, M.; Sturrock, S.; Buxton, S.; Cooper, A.; Markowitz, S.; Duran, C.; et al. Geneious Basic: An integrated and extendable desktop software platform for the organization and analysis of sequence data. *Bioinformatics* **2012**, *28*, 1647–1649.
173. Stamatakis, A. RAxML-VI-HPC: Maximum Likelihood-based phylogenetic analyses with thousands of taxa and mixed model. *Bioinformatics* **2006**, *22*, 2688–2690.
174. Stamatakis, A. RAxML version 8: A tool for phylogenetic analysis and post-analysis of large phylogenies. *Bioinformatics* **2014**, *30*, 1312–1313.
